# The Belt and Road Initiative, Public Health Expenditure and Economic Growth: Evidence from Quasi-Natural Experiments

**DOI:** 10.3390/ijerph192316234

**Published:** 2022-12-04

**Authors:** Xin Cao, Peng Li, Shi Li, Heng Zhang, Mengni Qin

**Affiliations:** 1 School of Economics, Guangxi University, Nanning 530004, China; 2 School of Economics and Trade, Guangxi University of Finance and Economics, Nanning 530003, China; 3 School of Business, Guangxi University, Nanning 530004, China; 4 School of Management, Lanzhou University, Lanzhou 730000, China

**Keywords:** the belt and road initiative, public health expenditure, economic growth, sustainable development, difference-in-differences

## Abstract

The United Nations 2030 Sustainable Development Goals (SDGs) involve society, economy, and environment, and the Belt and Road Initiative (BRI) is an important path to implement the SDGs. Moreover, the BRI is a vision for economic development of countries along the route. Although many studies documented the effect of the BRI on environment and economic performance, few studies have discussed the effect of the BRI on social and economic benefits. Therefore, we introduce the public health expenditure to explore the relationship between the BRI and the public health and economic growth of countries along the route from the dual perspective of social development and economic growth. Based on a panel data from 171 countries from 2010 to 2018, the current research explores whether the BRI can boost public health and promote economic growth in the belt-road countries. As a result, we found that the BRI boosted the expenditure of public health and effectively spurred economic growth in the belt-road countries. Furthermore, the effect of the BRI on the economic growth in the countries along the route depends on the level of public health expenditure in each country; the positive effect of the BRI on economic growth is significant when the public health expenditure level is moderate instead of low or high. The findings provide theoretical and practical insights into the SDGs of the BRI.

## 1. Introduction

The relationship between the Belt and Road Initiative (BRI) and sustainable development goals has long been an important topic in the literature [1,2]. However, few studies have discussed the effect of the BRI on social and economic benefits at the same time. Therefore, we introduce the public health expenditure to explore the relationship between the BRI and public health and economic growth from the dual perspectives of social development and economic growth. The BRI is committed to promoting economic growth through mutual cooperation in five areas: policy coordination, infrastructure connectivity, unimpeded trade, financial integration, and closer people-to-people ties [1,3,4]. While promoting the economic growth of each partner, the BRI also attaches great importance to the Sustainable Development Goals (SDGs) [1,2], and the “Guidance on Promoting a Green Belt and Road” issued by China in 2017 proposed the promotion of green Belt and Road construction and the joint achievement of the United Nations (UN) 2030 SDGs by countries and regions along the route.

The UN 2030 SDGs focus on society, economy, and environment, and the BRI is an important path to achieve the UN SDGs. The relationship between the BRI and sustainable development has been documented in many studies, including energy consumption and sustainable growth [1], the BRI and carbon emissions [5,6], the BRI and energy intensity [7], and the BRI and green total factor productivity [1,2]. These studies provide evidence on the relationship between the BRI and sustainable development [1,7]. However, these studies mainly discuss the relationship between the BRI and sustainable development from the perspective of the environment or economy, while the social dimension is limited. In fact, the UN SDGs emphasizes that sustainable development involves economic, social, and environmental aspects. Thus, we further investigated the effect of BRI on economic growth and public health to give more evidence in the research on sustainable development.

In the domain of society, human health is a fundamental social issue [8], and the classical theory of economics also takes health as an important human capital and one of the important factors driving economic growth [9,10]. In the “Outlined vision and proposed actions to jointly build the Silk Road Economic Belt and 21st-Century Maritime Silk Road” released in 2015, China mentioned that it would strengthen cooperation with neighboring countries on epidemic information sharing, the exchange of prevention and treatment technologies, the training of medical professionals, and improve the capability to jointly address public health emergencies. Promoting cooperation in public health among countries has been a major part of the BRI. Public health can increase personal income and economic output through an increase in the quality and quantity of workers, which can have an indirect effect on economic growth by influencing demographics and education [11,12,13]. Although most research on the relationship between the BRI and sustainable development has documented the effect of the BRI on the environmental and economic growth, few studies have discussed the effect of the BRI from the dual perspectives of social development and economic growth [3]. This research gap prevents us from better understanding the relationship between the BRI and sustainable development, thus, it is urgent for us to further examine the effect of BRI on public health and economic growth from social perspective [14]. Specifically, this study hopes to answer the following questions: (1) Does the BRI promote economic growth in BRI countries? (2) Does the BRI boost public health expenditure in countries along the route? (3) Does the positive effect of the BRI on economic growth in countries along the route depend on the level of public health expenditure level in each country? To do so, we integrated the social perspective to the relationship between the BRI and sustainable development in the context of the UN 2030 SDGs. Therefore, we contribute to the literature on the BRI from the social and economic perspectives by exploring the relationship between BRI and public health expenditure and economic growth.

Based on a panel data from 171 countries from 2010 to 2018, we constructed the difference-in-differences (DID) model to investigate the above questions. First, we used the DID to assess the effect of the BRI on economic growth in the belt-road countries. Second, we added the public health expenditure to explore the effect of the BRI on public health expenditure in the belt-road countries from the perspective of social development. Finally, we further explored under what circumstances the positive effect of the BRI on economic growth will occur by considering the level of public health expenditure in each belt-road country, which revealed how public health expenditure affects the relationship between the BRI and economic growth.

In summary, to contribute to the abovementioned research gaps, the current study integrated public health expenditure into the relationship between the BRI and economic growth in the belt-road countries to explore the effect of the BRI from the dual perspectives of social development and economic growth. The three research questions proposed in this paper involve the relationship between the BRI and economic growth, the BRI and public health expenditure, and how the public health expenditure level influences the relationship between the BRI and economic growth. Our research makes several important contributions. First, we included public health expenditure in the relationship between the BRI and economic growth and found that the BRI significantly boosts public health expenditure in countries along the route. As opposed to previous studies that focused on the environmental or economic dimensions to examine the relationship between the BRI and sustainable development [5,6], this paper discussed the relationship between the BRI and sustainable development from the dual perspectives of social development and economic growth, adding a new social perspective to the research on the relationship between the BRI and sustainable development. Second, this paper explored the relationship between the BRI and economic growth from the social perspective and specifically examined how public health influences the effect of the BRI on economic growth; we found that the public health expenditure in each country along the route can serve as a mechanism to explain the effect of the BRI on economic growth. Third, this paper assessed the effect of the BRI on economic growth, the effect of the BRI on public health expenditure, and how the public health expenditure influenced the effect of the BRI on economic growth by using the DID model. Compared to previous studies [1], this paper provides rich causal evidence for the relationship between the BRI and sustainable development using quasi-natural experiments.

## 2. Literature Review and Research Hypothesis

### 2.1. The BRI and Economic Growth

The BRI provides a vison for mutual cooperation in economic growth [1,15,16]. The vision involves international trade between partner countries, infrastructure and financial connectivity, policy integration and coordination, and the sharing of technology for the development and economic growth of partner countries [4]. The BRI has the potential to develop a unified world trade partnership and a strong common sense of cooperation including economy, politic, culture, and society that will lead to a better future for all partner countries [17]. Specifically, the BRI could exert a positive effect on the economic development of partner economies through trade expansion; access to advanced markets; sharing of skills, technology, and labor; and financial resources, which can flow into less developed and developing countries and emerging economies [18]. Moreover, there are many projects that include cooperation in infrastructure construction, such as business, industry, poverty alleviation, power generation, roads, and railroads, which will play an important role in driving economic growth in BRI countries [1]. Based on the above evidence, we believe that the BRI can effectively spur the economic growth in the belt-road countries and propose the following hypothesis:

**Hypothesis** **1** **(H1).** *The BRI effectively promoted the economic growth of BRI countries.*

### 2.2. The BRI and Public Health Expenditure

According to the triple bottom line theory, social benefit is an important component for sustainability [19]. The BRI is an effective avenue to promote social progress and is beneficial to achieve the 2030 SDGs. The Beijing Communique on “The Belt and Road” Health Cooperation and “Health Silk Road” (hereinafter referred to as Beijing Communique) released in 2017 puts forward the concept of building a “Health Silk Road”; the literature also takes the BRI as a health road [20,21]. Therefore, this section analyzes the effect of the BRI on social progress from the perspective of public health in three aspects., promoting public health through cooperation agreements between countries and international organizations; promoting public health expenditure through investment in health care and cooperation in disease and epidemic prevention and control; and promoting public health cooperation through social aid.

First, it is beneficial to promote public health through cooperation agreements between countries and international organizations. According to “The Belt and Road Initiative Progress, Contributions and Prospects” statistics released by China in 2019, China has strengthened health cooperation with BRI countries, signing 56 agreements with Mongolia and Afghanistan, international organizations such as the World Health Organization, and nongovernmental organizations (NGOs) such as the Bill and Melinda Gates Foundation to promote health cooperation. In 2017, the BRI and Health Silk Road High Level Symposium was held in Beijing, and the Beijing Communique was issued. China has established a public health partnership with the Lancang-Mekong countries, Central and West Asian countries, Association of Southeast Asian Nations (ASEAN) countries, and African countries. The signing of these cooperation agreements can promote public health expenditure and progression.

Second, it is useful to promote public health with investment in health care and cooperation in the field of disease and epidemic prevention and control. The BRI promotes global cooperation of public health in partner countries to cope with the threat of serious and new public health issues [22]. Specifically, the BRI can promote international cooperation to fight against infectious diseases. Previous research documented that the BRI could make progress in the prevention and elimination of infectious diseases, such as human immunodeficiency virus (HIV), tuberculosis (TB), malaria and acquired immunodeficiency syndrome (AIDS), and neglected tropical diseases (NTDs) [23]. During the COVID-19 outbreak, China’s efforts to fight against the epidemic and the international cooperation platform provided by the BRI have also played an important role in the prevention and control of COVID-19 [20]. For example, China has established overseas centers for traditional Chinese medicine (TCM) in 35 countries along the route and built 43 bases for international cooperation in TCM. Thus, it is evident that the public health expenditure will boost fast with the cooperation in the medical field and disease management.

Third, it is helpful to promote public health through social aid. According to “The Belt and Road Initiative Progress, Contributions and Prospects” statistics, the Chinese government will provide 2 billion RMB of emergency food aid to the belt-road countries, provide replenishment of US $1 billion to the South-South Cooperation Assistance Fund, and support the implementation of 100 Happy Home Projects, 100 Anti-Poverty Projects, 100 Health Recovery Projects, and other projects in the relevant countries. These social aids provide financial support to promote public health in the countries along the route.

Based on the above analysis, there is no doubt that the BRI could strengthen public health cooperation among countries along the route, and therefore, boost public health expenditures. Thus, the following hypothesis is proposed.

**Hypothesis** **2** **(H2).** *The BRI effectively promoted the public health expenditure of BRI countries.*

### 2.3. The Effect of Public Health Expenditure on the Relationship between the BRI and Economic Growth

The previous section assumes that the BRI promotes economic growth and public health expenditure in the belt-road countries. This section explores how public health expenditure in each country influences the impact of the BRI on economic growth. Although the BRI promotes economic growth, it also places great importance on sustainable development [3]. Thus, we discuss the relationship between the BRI and sustainable development at the country level, focusing on the relationship between social benefits and economic performance. We predict that the effect of the BRI on economic growth will depend on the levels of public health expenditure.

The relationship between health and economic growth has been one of the interesting issues in the domain of in sociology and economics. According to the endogenous growth theory, health has four effects on economic growth: health influences labor productivity and human capital accumulation by affecting educational returns; health can promote health investment caused by increased personal utility, and thus bring a crowding-out effect on physical capital; and finally, health may lead to population aging as the health levels improve [24]. The first two effects of health tend to promote economic growth, but the latter two may inhibit economic growth. On the one hand, excessive health investment will crowd out physical capital investment and thus exceed the level of health at optimal growth, making health investment inhibit economic growth. On the other hand, health investment of the elderly would lead to population aging, which then increases the share of the retirees in the total population, resulting in a reduction in labor and thus inhibiting economic growth [24,25]. Therefore, it is necessary to identity under what circumstances the effect of health on economy is positive [26].

Many empirical studies have also provided evidence for a complicated relationship between health and economic growth. Keely and Schmidt (1995) examined the relationship between health and economic growth from the mortality perspective and found an inverted U-shaped relationship [27]. To continue, the extant research has also confirmed that health may both promote and inhibit economic growth at the macro level [28,29]. Similarly, some research has shown a dual effect of health expenditure on economic growth from the perspective of health investment [30,31]. Thus, the following hypothesis is proposed:

**Hypothesis** **3** **(H3).** *The effect of the BRI on economic growth in belt-road countries depends on the level of health expenditure in each country. Specifically, the positive effect of the BRI on economic growth is most significant when a country has a moderate level of health expenditure instead of a low or high level.*

## 3. Research Design

### 3.1. Model Design

Considering that the DID is based on a counterfactual framework and can effectively estimate the treatment effect of exogenous policies (e.g., BRI), this paper takes the BRI as a quasi-natural experiment and used the DID for empirical tests to accurately evaluate the effect of the BRI on economic growth in the belt-road countries [32,33]. When the BRI was initiated in 2014, differences in the belt-road countries were captured from a time dimension and unit dimension simultaneously using the DID method.

Accordingly, we selected 2014 as the policy point of the BRI, while selecting the belt-road countries as the treatment group and non-belt-road countries as the control group [34]. Based on this, the DID model was constructed as follows:

(1) $${\mathrm{pgdp}}_{\mathrm{it}}=\alpha_{0}+\alpha_{1}\mathrm{dudt}+\gamma x+\lambda_{i}+\delta_{t}+\mu_{\mathrm{it}}$$

Formula (1) is a two-way fixed effects DID model, where ${\mathrm{pgdp}}_{\mathrm{it}}$ is the explained variable, indicating the level of economic growth in period t of country i. The du is the dummy variable for the treatment group, and dt is the time dummy variable. The dudt is the core explanatory variable, which is the interaction term between the time dummy variable and the treatment group dummy variable, and its coefficient $\alpha_{1}$ explains the effect of the BRI on economic growth. x represents the control variables. The $\lambda_{i}$ is the country fixed effect, $\delta_{t}$ is the time fixed effect, and $\mu_{\mathrm{it}}$ is a random error term.

### 3.2. Data and Variable Measurement

Considering that the effects of the BRI and lag phenomenon, we selected 2014 as the policy point of the BRI; therefore, we treated the 5-year period from 2014–2018 as the post-shock (BRI) period and the 4-year period from 2010–2013 as the pre-shock period to examine the effect of the BRI on the economic and social effects. This paper used panel data for 171 countries from 2010–2018 from the origin parameter-global macroeconomic database, including 60 belt-road countries and 110 non-belt-road countries. To reduce heteroskedasticity, the main variables are expressed by the natural logarithm. Additionally, to avoid the effect of extreme values on the results, we winsorized all continuous variables at the 1% and 99% quartiles.

The explained variable ${\mathrm{pgdp}}_{\mathrm{it}}$ is economic growth, which indicates the level of economic growth of country i in period t, measured by GDP per capita. We first set dummy variables for the treatment group and time dummy variables, where du is the dummy variable for the treatment group; if it is a belt-road country, then du takes the value of 1, otherwise, it takes 0. dt is the time dummy variable; the value of dt will be 1, if time is in 2014 (policy point) and after. Conversely, dt takes the value of 0 if time is before 2014. Second, dudt is the interaction term of du and dt. When country i belongs to the belt-road and in period t (year ≥ 2014), the value of dudt is 1, otherwise the value is 0. To continue, we referred to the existing literature [1,35] and selected control variables, including government size (gov), measured by government expenditure as a share of GDP; trade dependence (lnopen), measured by the natural logarithm of the economic freedom index for each country; education level (educ_1), measured by education expenditure as a share of GNI; and population in each country (lnpop), measured by the natural logarithm of each country. The results of descriptive statistics for the main variables are shown in Table 1.

## 4. Results and Discussion

### 4.1. Baseline Regression Results

In this section, we examine the effect of the BRI on the economic growth in the belt-road countries. To do so, the regression results of model (1) are reported in Table 2. The column (1) and column (2) are the regression results of the two-way fixed effects model, which controls country fixed effects and time fixed effects at the same time. Additionally, the column (1) is the result without adding control variables, while the column (2) is the result with control variables. The results both showed that the regression coefficients of dudt are significant at 5%. The result of column (2) showed that the BRI has a positive effect on per capita GDP in the belt-road countries at the 5% significance level (α=0.031,p<0.05), indicating that compared with non-belt-road countries, the average per capita GDP of a country along the route will increase by 3.1%. Therefore, H1 is supported.

### 4.2. Parallel Trend Test

To further ensure the validity and accuracy of the baseline regression results, we referred to the method of [34,36] to examine the parallel trends between the treatment group and control group. In this paper, 2014 is taken as the year of the BRI announcement, so the time dummy variable of the 3 years before the BRI and the 4 years after the BRI are defined to examine the trends.

**Figure 1:**
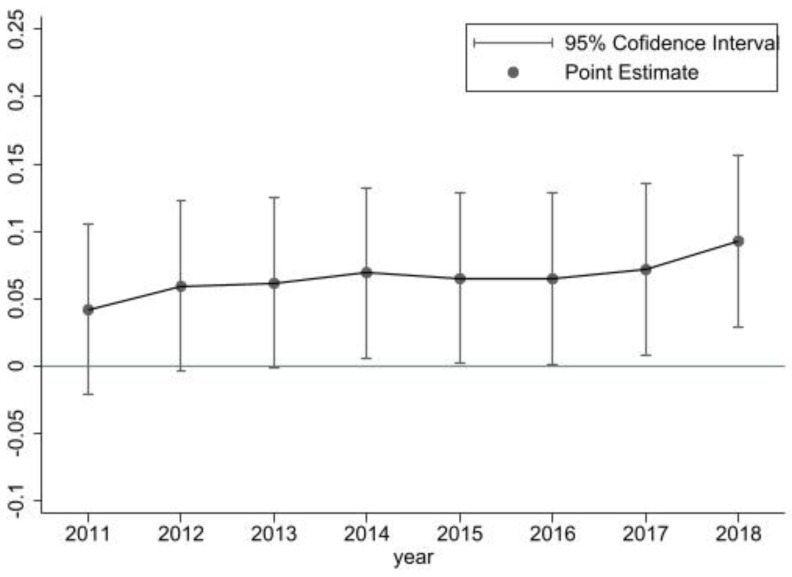
Results of the parallel trend test.

In Figure 1, the vertical axis represents the estimated interaction term between the experimental group and different years, which evaluates the dynamic economic growth effects for each year before and after the BRI, and the horizontal axis represents the number of years before and after the BRI. Moreover, the estimated coefficients of interaction terms are not significant, indicating that the trend of the belt-road countries and non-belt-road countries has no system difference before the BRI (2014), which proves that the DID method is valid and reliable. In and after 2014, the estimated coefficients of the interaction term are significantly positive and become larger, which means that the economic effect of the BRI appears after 2014. Taken together, our main results that the BRI can significantly promote economic growth are robust and reliable.

### 4.3. Robustness Test

#### 4.3.1. Placebo Test

Moreover, we referred to the method of Yang et al. to conduct a placebo test by randomly selecting the number of countries in the treatment group from the sample [37] to exclude the effect from the unobservable factors on our baseline results. Specifically, the sample of this paper contains 171 countries, including 61 belt-road countries. Accordingly, 61 countries were randomly selected from the sample of 171 and set as the “false” treatment group, and the remaining 110 countries were considered as the “false” control group to construct a dummy variable ${\mathrm{du}}_{i}^{\mathrm{false}}$ and then construct interaction term ${\mathrm{du}}_{i}^{\mathrm{false}}\times {\mathrm{dt}}_{t\;}$. Given that the “false” experiment group is randomly generated, the coefficients of interaction terms would not deviate significantly from zero, which indicates that the promotion of economic growth in belt-road countries is caused by the implementation of the BRI, rather than other unobservable variables. Additionally, the above procedure was repeated 500 times to exclude the confusion from the effect of other small probability events on baseline results.

**Figure 2:**
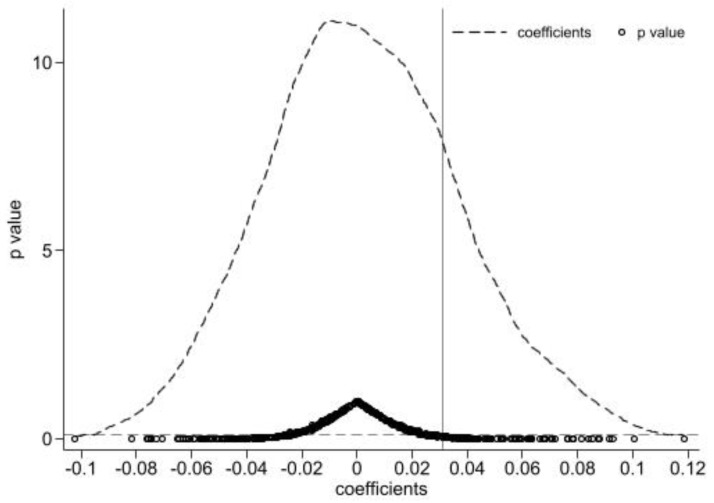
The result of the placebo test. Note: The vertical line on the left side of the figure represents the actual estimated coefficient. The horizontal dashed line at the bottom of the figure represents *p* = 0.1.

As shown in Figure 2, the mean values of the estimated coefficients are close to 0, and most of the p values are greater than 0.1, which means that most of the estimated coefficients do not deviate from zero statistically. In addition, the vertical line, which refers to the actual estimated coefficients, accounts for a small proportion in the distribution of estimated coefficients and are outliers clearly. Based on the above analysis, it can be inferred that the promotion of economic growth is really caused by the BRI. Therefore, it is reasonable to believe that the core conclusions are very robust.

#### 4.3.2. PSM-DID

Furthermore, we referred to the method of Sun and Li, to use the PSM-DID method, which integrates the propensity score matching and the DID, to better exclude the effect of sample selection [32] bias on the baseline results. Specifically, based on the kernel matching method, we estimated the propensity score for each belt-road country through a Logit regression model, in which the explained variable was whether the country is a belt-road country and the covariates were the above control variables.

Additionally, the changes in covariates between the treatment group and control group before and after matching are reported in Table 3. It can be inferred that the differences in characteristic covariates among countries after matching are not significant at the 10% level, indicating that PSM significantly reduced intercountry differences. Moreover, the percentage bias between the belt-road countries and the non-belt-road countries was significantly reduced after matching [38]. In summary, the balance test of the PSM method is valid.

**Figure 3:**
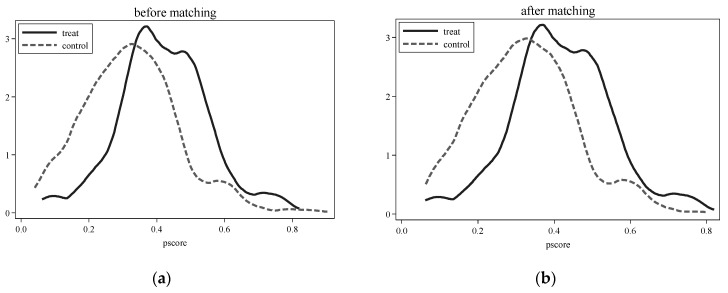
Probability distribution density function of tendency score value.

Second, the propensity score value density function of the PSM was plotted (see Figure 3a,b); the propensity score value probability density distributions of the samples in the belt-road countries and non-belt-road countries are closer after kernel matching, which further verifies that the matching effect is better. Thus, based on the matched samples, the baseline model was regressed again (see column (1) of Table 4). The result showed that the coefficient of dudt was significantly positive at the 5% level (α=0.033,p<0.05), which again verifies that compared with non-belt-road countries, the average per capita GDP of a country along the route will increase by 3.3%. In conclusion, after solving the threat of selective bias existing in the sample, the estimated results in this paper are still robust and stable.

#### 4.3.3. Dropping Samples in 2013

The existing research considered 2010–2013 as the period before the BRI and 2014–2018 as the period after the BRI implementation. The BRI was first introduced in 2013, and in 2013, the global economy gradually recovered and some stimulating economic policies were introduced to promote economic growth, such as quantitative easing money policy, tax and fee reduction fiscal policy, and structural reform, which may pose a threat to our results; therefore, we dropped the sample in 2013 to enhance the robustness of our results. Then, we put the remaining sample into the baseline model for regression. The results are reported in Table 4.

It can be seen from column (2) in Table 4 that the regression coefficient of dudt is significantly positive at the 5% significance level (α=0.039,p<0.05), indicating that the economic policy in 2013 and the pilot year of BRI (2013) did not have a significant impact on the economic growth promotion effect of the BRI. Accordingly, the BRI still had a significant effect on promoting economic growth, which again shows the robustness of the estimated results in this paper.

#### 4.3.4. Dropping Samples in America

The countries covered in our sample include seven regions: the East Asia and Pacific, Europe and Central Asia, Latin America and Caribbean, Middle East and North Africa, North America, and South Asia and Sub-Saharan Africa. It should be noted that the Latin America and Caribbean and North America regions are far away from China and differ greatly from other regions. Furthermore, none of the countries in the two regions are included in the Belt and Road list. To avoid the effect of these two specific regions on the estimation of the results, we referred to the method of Song et al. (2021) to exclude these two regions from the sample for robustness analysis [39]. Then, we put the remaining sample into the baseline model for regression. The results are reported in column (3) of Table 4.

It can be seen from column (3) in Table 4 that the regression coefficient of dudt is significantly positive at the 1% significance level (α=0.209,p<0.01), indicating that the BRI has a significant effect on promoting economic growth in the belt-road countries even when excluding the effect of the two specific areas. Therefore, the core result in this paper is reliable.

Based on the above analysis, we consistently find that the BRI is effective to promote economic growth in the belt-road countries; this result is consistent with the findings of existing research [4]. The BRI activates the dynamics of economy through the mechanism of unimpeded trade, policy coordination, financial integration, facilities connectivity, and people-to-people bonding to promote economic growth in the countries along the route [40]. With the economic cooperation in partner countries strengthened since the announcement of the BRI [2], the economic achievements are gradually appearing, which reflects the facts of economic development for the BRI. In this paper, we empirically tested this conclusion and provide robust evidence.

### 4.4. Heterogeneity Test

#### 4.4.1. Distinction between “the Belt” and “the Road” Countries

The Belt and Road refers to the “Silk Road Economic Belt” and the “21st Century Maritime Silk Road”. Given that the focus of the BRI is the level of economic development, historical, and cultural factors in belt-road countries, it is necessary to divide the belt-road countries into the belt and the road countries [34], and construct the interaction terms du_dt_belt (du × dt × belt) and du_dt_road (du × dt × road) and put them into the baseline model for regression. The results are reported in Table 5.

The regression results are reported in columns (1) and (2) in Table 5. The regression coefficients of the interaction term du_dt_belt was significantly positive (α=0.073,p<0.01), indicating that the BRI can significantly promote the economic growth in the belt countries; however, this effect was not significant in the road countries (α=−0.004,p>0.05). This shows that the BRI has significant regional heterogeneity in promoting economic growth. The reason may be a difference in Chinese companies’ investment in infrastructure in the belt-road countries [34]. The belt countries mainly include countries in Central Asia, West Asia, and the east coast of Africa, which have poorer infrastructure for economic development than the road countries. Meanwhile, the development of infrastructure plays a central role in the BRI. Thus, the belt countries have a great potential to benefit from the investment of infrastructure construction and then promote the economic growth significantly. However, the road countries may have a high-level economic cooperation and be in a period of investment, thus, there is a time lag in the effect of economic growth.

#### 4.4.2. Distinction between “Near” and “Non-Near” Countries

Considering that geographical distance is an important factor influencing economic trade between countries, prior research has explored differences in the impact of geographical distance on transportation investment in countries along the route [41]. This paper divides the belt-road countries by geographical distance. The belt-road countries were classified into “near” and “non-near” countries according to their geographical distance from China; the interaction terms du_dt_near (du × dt × near) and du_dt_non_near (du × dt × non-near) were constructed and put into the baseline model for regression.

The results are shown in Table 6. Quite clearly, the estimated coefficient of the du_dt_near was significantly positive (α=0.208,p<0.01), indicating that the BRI can significantly promote economic growth in the near countries, but this effect is not significant in the non-near countries (α=−0.015,p>0.05). The reason may be that China and its near countries are more harmonious and less geopolitically risky in terms of facility connection, policy communication, and financial integration [42]. In addition, the increasing institutional differences occur due to the increase in geographic distance from China [41]. Institutional differences between the host and home countries are important factors affecting outward investment for firms [43]. Additionally, geographic distance may be a key variable affecting institutional diffusion; the greater the geographic distance from China, the greater the difference in institutional distance from China is likely to be. Thus, geographical distance may affect Chinese firms’ outward investments, which influences institutional distance. In addition, the Chinese firms’ may prefer to invest in the near-countries than non-near countries. Therefore, the BRI has a more significant promotional effect on near countries.

#### 4.4.3. Distinguishing between Low-Income Countries and Others

The purpose of the BRI is to promote regional economic development by creating win-win cooperation and joint prosperity. As an important part of “infrastructure connectivity”, infrastructure development has been a priority and a key driver of economic growth for the BRI [40]. For countries with relatively backward economies, improving infrastructure construction is a key area to promote economic growth in the process of the BRI. Therefore, this paper also focuses on whether the BRI promotes economic growth in low-income countries. Based on the level of economic development, this paper classified countries into “low-income countries” and “other countries”, and then performed grouping regression.

The results are reported in Table 7, which shows that the BRI significantly promoted economic growth in low-income countries (α=0.119,p<0.01), but it was significantly negative for “other countries”. The reason may be that the BRI spared no efforts to promote the economic growth of developing countries and low-income countries through infrastructure development, unimpeded trade policy coordination, and financial integration. Thus, the BRI is more attractive and receives a positive response in low-income countries. Accordingly, the BRI has a significantly positive effect on economic growth in low-income countries. In addition, for other countries with higher levels of economic development, cooperation is not limited to infrastructure but also requires high technology and huge investment; the diversity of investment may carry risk and uncertainty, which increases the complexity of the impact on economic growth. Thus, the BRI was significantly negative for “other countries”.

## 5. Mechanism Analysis

As described above, the effect of health on economic growth is complicated. Although we have provided robust evidence that the BRI has a significantly positive effect on economic growth, it is not clear that whether the BRI can boost public health and how the relationship between BRI and economic growth will change when adding public health expenditure to the analysis. Meanwhile, this paper predicts that the BRI will boost the public health and that the effect of the BRI on the economic growth of BRI countries depends on the public health expenditure level in belt-road countries. To examine the role of public health expenditure in the BRI and economic growth, we first took public health expenditure as an explanatory variable and used the DID method to estimate whether the BRI promoted public health in countries along the route, and test H2. Second, we classified the public health expenditure into three groups, low, medium, and high, and used the baseline model to perform regression for each group to test how the effect of the BRI on economic growth depends on the different levels of public health, and to test H3.

### 5.1. The Impact of the BRI on Public Health Expenditure along the Route

In this section, public health expenditure is the explanatory variable. The existing research documented that in consideration of the proxy variable of public health, there may be some problems such as data quality and exchange rates, causing the absolute value of government expenditures to be not comparable, in general; the share of health expenditure in GDP can be used as a proxy variable for health expenditures [31,44]. Thus, this paper took the share of health care expenditure in GDP of each country as the proxy variable for public health expenditure (phexp). Similarly, we used the DID model with two-way fixed effects, controlling the country fixed effect and year fixed effect at the same time. The estimated results are presented in Table 8.

The results show that the regression coefficients of the dudt are all significant at 5%. The regression result of column (2) shows that the BRI had a positive effect on public health in the belt-road countries at the 5% significance level (α=0.001,p<0.05), indicating that compared with non-belt-road countries, the average public health of a country along the route will be promoted by 0.1%. Therefore, H2 is supported. This result is consistent with the current reality of cooperation with countries along the route in the field of public health and reflects the efforts for the construction of Health Silk Road [18]. As discussed above, the BRI is an effective cooperation platform for partners to copy with the challenge in the field of public health [22], such as the prevention and elimination of infection diseases [23]. Therefore, with cooperation in the field of public health strengthening and deepening, the investment and expenditure in public health is increasing.

The BRI also attaches great importance to the social progress in the belt-road countries; public health is an important component of social progress. In addition, previous research on the relationship between the BRI and sustainable development has mainly discussed the relationship between the environment and the economy [4,6], with few studies considering the social dimension. Thus, we extended this research area by adding a new perspective to the study of the relationship between the BRI and sustainable development from the country level. Specifically, we introduced public health expenditure from a social perspective to test the effect of the BRI on public health in belt-road countries. To do so, this paper did not simply assess the economic effects of the BRI but rather further investigated effect of the BRI on public health. The above results show that the BRI has made substantial progress in cooperation in public health. According to “The Belt and Road Initiative Progress, Contributions and Prospects” released in 2019, China has cooperated with several countries along the route in the field of health care.

### 5.2. The Effect of Public Health on the Relationship between the BRI and Economic Growth

To examine how the effect of the BRI on economic growth depends on the level of public health expenditure, we further classified the public health expenditure by quartiles (33rd and 66th quartiles) into three groups, low, medium, and high public health expenditure, and performed baseline regressions for each group to test H3. Considering the data used in this paper is panel data, we use Fisher’s permutation test with the number of self-samplings set to 1000 to test whether there was a significant difference in the estimated coefficients of the regressions for each group.

The results of the regressions for three groups are reported in Table 9. Column (1) is the estimated results for the low public health expenditure group; the result shows that the effect of the BRI on economic growth is not significant (α=0.005,p>0.1). Column (2) is the estimated results for the medium public health expenditure group; the result shows that the effect of the BRI on economic growth is significantly positive (α=0.113,p<0.01). Column (3) is the estimated results for the high public health expenditure group; the result shows that the effect of the BRI on economic growth is significantly negative (α=−0.054,p<0.05).

Furthermore, Fisher’s permutation test was applied to test for differences in estimated coefficients between the three groups. The results also showed that the coefficient difference was 0.05 (*p* < 0.001) between the medium group and the low group; the coefficient difference was 0.059 (*p* < 0.001) between the high group and the low group; and the coefficient difference was 0.167 (*p* < 0.001) between the medium group and the high group. Taken together, it can be inferred that the relationship between the BRI and economic growth depends on the level of public health expenditure. Specially, the effect of the BRI on economic growth is significantly positive at a medium level of public health expenditure; however, the effect of the BRI on economic growth is significantly negative at the high level of public health expenditure. Thus, we identified that the relationship between BRI and economic growth is influenced by the level of public health expenditure. In the different public health expenditure conditions (low vs. medium vs. high), the effect of BRI on economic growth was significantly different. Once the public health expenditure is too much, it may exert a negative effect on the economic growth. In summary, H3 is supported.

Previous research has documented that the relationship between health and economic growth is complicated; most studies used qualitative analyses [45,46] to analyze their relationship. We extend this research area by further identifying that the BRI had the most significant effect on economic growth when public health expenditure was at medium level rather than a low or high level with the DID method. This finding is consistent with the existing literature on the relationship between health and economic growth [26], and confirms the endogenous growth theory about the four effects that health will have on economic growth [24,25]. When the level of public health expenditure is low, the effect of health investment mainly improves health through increased nutrition, and the effect of economic promotion is not obvious [10]. When public health expenditure is at a moderate level, health contributes to economic growth by increasing the capabilities of the workforce and influencing the returns to education [24,25]. When public health expenditure is at a high level, health may have a dampening effect on economic growth by crowding out physical capital investment and population aging [24,25]. Therefore, when we analyze the relationship between the BRI and economic growth from the perspective of sustainable development, we need to seek different ways of cooperation according to the development stage of public health expenditure in each country.

## 6. Conclusions and Policy Recommendations

At present, there are limited studies exploring the relationship between the BRI and economic growth from the dual perspectives of social progress and economic growth under the background of Health Silk Road from the country level. Therefore, based on the panel data of 171 countries from 2010 to 2018, the current research took the BRI as a “Quasi-natural Experiment” and constructed a two-way fixed effects DID model to explore whether the BRI can boost public health and promote economic growth in the belt-road countries. First, we found that the BRI effectively promoted the economic growth in the belt-road countries. The average per capita GDP of a country along the route increased by 3.1%. This result is consistent with the findings of existing studies [4] and in fact reflects the reality of the international cooperation since the BRI was launched. Importantly, in consideration of a series of robustness tests, including placebo tests, PSM-DID tests, dropping the sample in 2013, and dropping sample in America, our core conclusion remains robust and stable. Second, in the section on the heterogeneity analysis, we found that the promotion of economic growth in the countries along the route was mainly reflected in the belt countries. This result may be related to the difference in Chinese companies’ investment in infrastructure in BRI countries [34]. Additionally, the BRI significantly promoted the economic growth in the near countries; however, this effect was not significant in the non-near countries, which may be related to the increase in institutional differences due to the increase in geographic distance [37]. Furthermore, the BRI had a more significant effect on economic growth in low-income countries, which may be related to the type of Chinese investment in countries along the route. Third, the BRI effectively promoted public health in the belt-road countries. Specifically, compared with non-belt-road countries, the average public health expenditure of a country along the route was promoted by 0.1%. Fourth, we found that the relationship between the BRI and economic growth depends on the level of public health expenditure. Specially, the effect of the BRI on economic growth was most significantly positive at the medium level of public health expenditure instead of the low and high levels.

Based on the above findings, we can provide the following policy suggestions.

First, the BRI effectively promotes economic growth in the belt-road countries. Therefore, policy makers in the belt-road countries should continue to vigorously promote the implementation of the BRI and strengthen the cooperation of partners to increase efforts to play the role of BRI in the economic growth. As the economic effectiveness of BRI is proved, the world should promote economic growth through the platform provided by the BRI.

Second, the BRI has also contributed to the public health expenditure of BRI countries, which is consistent with the concept of sustainable Belt and Road development; this suggests that the belt-road countries need to pay attention to both economic performance and social development in the process of the BRI. In the area of public health, an important goal of the BRI is to strengthen health cooperation among countries along the route, integrate the fragmented health governance mechanisms along the route, and create a more holistic, networked, multi-bilateral regional health cooperation structure. The formation of a stable and effective health governance mechanism will help improve the transparency, participation, and legitimacy of the construction of the Health Silk Road. This requires the belt-road countries to actively develop health partnerships, practice multilateralism in the process of institution construction, try to include multiple actors and issues, and establish a multi-governance cooperation mechanism in the belt-road countries, relevant international organizations, nongovernmental organizations, and market players. The cooperation in country levels will exert profound effects on social development.

Third, the effect of the BRI on economic growth depends on the level of public health expenditure in the belt-road country. Once the public health expenditure is too much, it may hamper the economic growth. How to balance the relationship between economic growth and public health expenditure is an important issue in the process of achieving the goal of UN 2030 SDGs. Thus, the policy suggestion is that it is necessary to construct a diversified public health cooperation pattern according to economic development level and the public health expenditure level in the belt-road countries. Specifically, in countries and regions where public health expenditures are relatively low, usually at lower levels of economic development, cooperation with these countries and regions focuses primarily on the health consumption and nutrition. Countries and regions with relatively moderate level of public health expenditures are typically in a stage of economic transition from underdevelopment to development, where health may have important implications for investment in education, fertility, and demographics; thus, collaboration during this stage can focus on the role of public health in economic and demographic transitions. When public health expenditure is at a relatively high level, the economy has usually reached a relatively high stage of development, where health affects the economy through its impact on life expectancy and the crowding-out effect of health expenditure on physical capital accumulation; thus, cooperation at this stage, on the other hand, should not be limited to economic growth but should focus on the issue of economic development quality.

Fourth, evidence from this study suggests that cooperation in public health between China and the belt-road countries has been well established. The COVID-19 pandemic and global public health emergencies have affected the public health of all partners along the route. COVID-19 has made countries more clearly understand that health is the core of development, a prerequisite and result of development, and an effective indicator of sustainable development. Therefore, developing and maintaining the vitality of the health systems of the countries along the route and promoting cooperation in the field of public health will not only help to promote people’s health, but also contribute to the promotion of economic development. Meanwhile, it is noteworthy that once public health expenditure is invested too high, it may have potential to hamper the economic development. How to balance the relationship between economic growth and public health expenditure is a major issue in the context of COVID-19. Thus, the policy makers should deepen cooperation in the BRI in response to epidemics and public health emergencies and strive to promote economic growth.

There are also limitations to the study. First, this paper focuses on the relationship between the BRI and public health and economic growth; however, it does not consider environmental factors. According to the UN 2030 SDGs, sustainable development includes economic, social, and environmental factors, and future research can integrate all three dimensions of sustainable development into the analysis simultaneously. Second, this paper focuses on the relationship between public health expenditure and economic growth under the background of the BRI and examines the difference in the effect of the BRI on economic growth when considering different levels of public health expenditure. The relationship between public health and economic growth is very complex, and future research can further analyze the mechanism of public health impact on economic growth in the context of the BRI. Third, this paper found that the effect of the BRI on economic growth differs between “the belt” and “the road”, “near” and “non-near”, and low-income countries and other countries in the heterogeneity test. However, the reasons for the differences are not discussed in depth, and future research could focus on the heterogeneity of the effect of the BRI on economic growth.

## Figures and Tables

**Table 1:** Descriptive statistical analysis (N = 1491).

Variables	(1)	(2)	(3)	(4)	(5)
	Define	Mean	Sd	Min	Max
du	Treatment group dummy variable	0.357	0.479	0.000	1.000
dt	Time dummy variable	0.555	0.497	0.000	1.000
dudt	The interaction term dudt	0.199	0.400	0.000	1.000
lnpgdp	GDP per capita in logarithm	11.670	2.322	7.107	17.651
educ_1	Ratio of education expenditure to GNI	0.042	0.018	0.010	0.099
gov	Ratio of government expenditure of GDP	0.321	0.130	0.126	0.861
lnopen	Logarithm of economic freedom index	4.098	0.181	3.642	4.757
lnpop	Logarithm of population	15.890	1.876	11.541	20.965

**Table 2:** The baseline regression results.

Variables	(1)	(2)
	Lnpgdp	Lnpgdp
dudt	0.039 **	0.031 **
	(0.016)	(0.016)
Constant	11.668 ***	15.091 ***
	(0.005)	(2.562)
Observations	1500	1500
R-squared	0.9966	0.9968
Country FE	Yes	Yes
Year FE	Yes	Yes
Controls	No	Yes

Note: Robust standard errors are reported in parentheses. ***,** represent significance levels of 1%, 5%, respectively. The following tables are the same.

**Table 3:** Balance test results of covariates.

Variable	Unmatched	Mean		%bias	*t*-Test	
	Matched	Treated	Control		*t*	*p*
educ_1	U	0.038	0.045	−42.4	−7.68	0.000
	M	0.038	0.038	−4.2	−0.74	0.458
gov	U	0.334	0.315	14.6	2.72	0.007
	M	0.332	0.332	−0.4	−0.06	0.949
lnopen	U	4.109	4.092	9.8	1.81	0.070
	M	4.111	4.118	−4.1	−0.61	0.543
lnpop	U	16.293	15.666	34.3	6.27	0.000
	M	16.328	16.435	−5.9	−1.03	0.305

**Table 4:** Regression results for robustness test.

Variables	(1) Psm	(2) Dropping Sample in 2013	(3) Dropping Sample in America
	Lnpgdp	Lnpgdp	Lnpgdp
dudt	0.033 **	0.039 **	0.209 ***
	(0.016)	(0.019)	(0.019)
Constant	14.649 ***	14.698 ***	3.925 ***
	(2.603)	(2.648)	(3.409)
Observations	1452	1334	1084
R-squared	0.997	0.997	0.997
Country FE	Yes	Yes	Yes
Year FE	Yes	Yes	Yes
Controls	Yes	Yes	Yes

Note: Robust standard errors are reported in parentheses. ***, ** represent significance levels of 1%, 5%, respectively.

**Table 5:** The distinction of the Belt and the Road.

Variables	(1) The Road	(2) The Belt
	lnpgdp	lnpgdp
du_dt_road	−0.004	
	(0.023)	
du_dt_belt		0.073 ***
		(0.018)
Constant	15.431 ***	6.746 ***
	(3.143)	(2.839)
Observations	1192	1300
R-squared	0.997	0.997
Country FE	Yes	Yes
Year FE	Yes	Yes
Controls	Yes	Yes

Note: Robust standard errors are reported in parentheses. *** represent significance levels of 1%.

**Table 6:** The distinction of near and non-near countries.

Variables	(1) Near	(2) Non-Near
	lnpgdp	lnpgdp
du_dt_near	0.208 ***	
	(0.020)	
du_dt_non_near		−0.015
		(0.017)
Constant	5.637 ***	15.494 ***
	(3.129)	(2.505)
Observations	1268	1438
R-squared	0.997	0.997
Country FE	Yes	Yes
Year FE	Yes	Yes
Controls	Yes	Yes

Note: Robust standard errors are reported in parentheses. *** represent significance levels of 1%.

**Table 7:** Distinguishing between “low income” and “other countries”.

Variables	(1) Low-Income Countries	(2) Other Countries
	Lnpgdp	Lnpgdp
dudt	0.093 ***	−0.059 ***
	(0.018)	(0.020)
Constant	22.550 ***	17.965 ***
	(3.469)	(3.331)
Observations	975	525
R-squared	0.997	0.998
Country FE	Yes	Yes
Year FE	Yes	Yes
Controls	Yes	Yes

Note: Robust standard errors are reported in parentheses. *** represent significance levels of 1%.

**Table 8:** The impact of the BRI on public health in the belt-road countries.

Variables	(1)	(2)
	Phexp	Phexp
dudt	0.002 **	0.002 **
	(0.001)	(0.001)
Constant	0.063 ***	0.072 ***
	(0.000)	(0.094)
Observations	1500	1500
R-squared	0.943	0.949
Country FE	Yes	Yes
Year FE	Yes	Yes
Controls	No	Yes

Note: Robust standard errors are reported in parentheses. ***, ** represent significance levels of 1%, 5%, respectively.

**Table 9:** Regression results for the three groups.

	(1)	(2)	(3)
	Group 1 (Low)	Group 2 (Med)	Group 3 (High)
dudt	0.005	0.113 ***	−0.054 **
	(0.030)	(3.20)	(0.025)
Constant	38.180 ***	2.196	2.691
	(4.586)	(4.479)	(4.260)
N	491	491	495
Country FE	Yes	Yes	Yes
Year FE	Yes	Yes	Yes
Controls	Yes	Yes	Yes
R-squared	0.996	0.998	0.998

Note: *t* statistics in parentheses; ** *p* < 0.05, *** *p* < 0.01.

## Data Availability

The raw data supporting the conclusions of this article will be made available by the authors, without undue reservation.
